# Band Structure Simulations of the Photoinduced Changes in the MgB_2_:Cr Films

**DOI:** 10.3390/nano5020541

**Published:** 2015-04-02

**Authors:** Iwan V. Kityk, Anatolii O. Fedorchuk, Katarzyna Ozga, Nasser S. AlZayed

**Affiliations:** 1 Faculty of Electrical Engineering, Czestochowa University of Technology, Armii Krajowej Street 17, 42-200 Czestochowa, Poland; E-Mail: cate.ozga@wp.pl; 2 Department of Inorganic and Organic Chemistry, Lviv National University of Veterinary Medicine and Biotechnologies, Pekarska Street 50, 79010 Lviv, Ukraine; E-Mail: ft@ua.fm; 3 Physics and Astronomy Department, College of Science, King Saud University, P.O. Box 2455, Riyadh 11451, Saudi Arabia; E-Mail: nalzayed@ksu.edu.sa

**Keywords:** nanocrystalline superconducting films, photoinduced treatment, MgB_2_

## Abstract

An approach for description of the photoinduced nonlinear optical effects in the superconducting MgB_2_:Cr_2_O_3_ nanocrystalline film is proposed. It includes the molecular dynamics step-by-step optimization of the two separate crystalline phases. The principal role for the photoinduced nonlinear optical properties plays nanointerface between the two phases. The first modified layers possess a form of slightly modified perfect crystalline structure. The next layer is added to the perfect crystalline structure and the iteration procedure is repeated for the next layer. The total energy here is considered as a varied parameter. To avoid potential jumps on the borders we have carried out additional derivative procedure.

## 1. Introduction

The possibility of operation by superconducting and non-linear optical properties of high temperature superconductors was reported in several reports [1,2,3]. This effect has an important influence on the further application of such superconductors and in particular for creation of laser operated technology within a temperature range of the superconductivity [4]. However, the obtained results seem to be prevailingly empirical and detailed mechanisms explaining the observed phenomena are not clear. One of the possible approaches for further studies is to perform the photoinduced studies of the superconducting nanocrystalline films containing MgB_2_ and Cr_2_O_3_ [5]. Here we have coexistence of two different crystalline phases, and the role of the corresponding interface may be crucial. Only the simulations which include both first principle band structure calculation and molecular dynamics simulations may clarify the origin of the effects. This is important for the material science engineering during the search for compounds with the desired non-linear optical properties.

In the present work we have performed the complex DFT band structure calculations for the MgB_2_:Cr_2_O_3_ nanocrystalline films applying the norm-conserving pseudopotential method [6]. All the simulations took into account a contribution of the phonon subsystem [7]. We simulated the nonlinear optical dispersion of the photoinduced changes for the band structure dispersion and the related nonlinear optical susceptibilities. The calculations were done using originally developed cluster procedure. The cluster sbetween the two substructures were optimized with respect to the minimum of total energy [8].

Initially, the simulations of the band structure were done for the perfect MgB_2_ crystals. Afterwards, the changes due to incorporation of the Cr_2_O_3_ were considered. Additionally, the bicolor laser beams effective dc-electric field strength was superimposed. The experimental procedure was similar to that presented in the references [9]. The whole procedure was finally verified by dispersion of the second order susceptibilities defining the corresponding values that were compared to the experiment.

## 2. Theoretical Calculations

### 2.1. Band Structure Calculation Method

The norm-conserving pseudopotential was chosen in a form proposed in the reference [6]:

(1) $$V_{ps}^{\left(\text{β},l\right)}=\sum_{i=1}^{3}\left[A_{i}^{\text{β}}+r^{2}A_{i+3}^{\text{β}}\exp(-{\text{α}}_{l}^{\left(l,\text{β}\right)}r^{2})\right]$$

where *A_i_*, *A_i+3_*, α*_l_*^(*l*, β)^ are fitting pseudopotential parameters for the ions of β kind and for the combination of orbital with angular momentum *l*. We solved a secular equation corresponding to a norm-conserving pseudopotential expressed by Equation (2):

(2) $$\|\left[h^{2}{\left(k+G_{n}\right)}^{2}/2m-E\left(k\right)\right]\delta_{n,n^{'}}+\sum V_{\alpha }\left(G_{n}^{'}-G_{n}\right)S_{\alpha }\left(G_{n}^{'}-G_{n}\right)\|=0$$

where *E*(*k*) is an eigen-energy dispersion for a ***k***-BZ point; ***G*_n_'**, ***G*_n_** are wave-vectors of interacting plane waves. Structural form-factors for the β*-th* kind of atoms were written in a form:

(3) $$S_{\beta }\left(G_{n}^{'}-G_{n}\right)=g^{\left(\beta \right)}/\Omega N_{a}\sum \exp\left[-i\left(G_{n}^{'}-G_{n}\right)\tau_{\beta }\right]$$

Here *g*^(β)^ is a weighting factor determining partial contributions of the reconstructed atomic positions during their successive replacement for perfect MgB_2_ crystalline structure. The same procedure is done during the addition of Cr_2_O_3_. Varying the weighting factor *g*^(β)^ we were able to operate by degree of modifications for the perfect MgB_2_. The latter factor is particularly important for the calculation of the basic matrix renormalized by different imperfections [10].

This method was verified earlier for different disordered materials and partially ordered solids including non-stoichiometric SiC [11]. A plane wave basis set containing a couple of plane waves was varied from several hundreds up to 3000 plane waves to achieve eigen-energy convergence equal to about 0.01 eV.

The solution (diagonalization) of the secular equation set (Equation (2)) was done by applying a modified Jacobi method QL [12]. Additional plane waves from an extended Lowdin basis set were included during the calculations. A Fourier transformed pseudopotential finally was in a form:

(4) $$V_{\alpha }\left(G_{n}^{'}-G_{n}\right)=1/\Omega \int V_{\alpha }\left(r\right)\exp\left[-i\left(G_{n}^{'}-G_{n}\right)r\right]$$

Electron screening effects were evaluated using a parameterized Perdew-Zunger screening potential expression in a form:

(5) $$\begin{array}{l} {\text{μ}}_{xc}=-0.6193/r_{s}-0.14392/(1+1.0529r_{s}^{1/2}+0.3334r_{s})\\ \left\{1+\left[\left(0.526r_{s}^{1/2}+0.3334r_{s}\right)/\left(3\left(1+1.0529r_{s}^{1/2}+0.3334r_{s}\right)\right)\right]\right\}\;\;\;\text{for}\;\;r_{s}>1\\ \mu_{xc}=-0.6193/r_{s}+0.031\ln\left(r_{s}\right)-0.0583\;\;\;\text{for}\;\;r_{s}<1 \end{array}$$

where *r_s_* = [3/(4πρ)]^1/3^ and ρ is electronic charge density space distribution. The traditional numerical Chadhi-Cohen method of special point was applied for calculation of space electron charge density distribution. Diagonalization procedure for determination of eigen-energies and eigen-vectors was carried out at special weighting points of the BZ for each reconstructed factors.

Acceleration of the iteration convergence was obtained by transferring 70%–85% of the (m − 1)-*th* iteration result to the m-*th* iteration. The following condition was taken as a criterion for self-consistency:

(6) $$\left|\left({\text{ρ}}_{m}-{\text{ρ}}_{m-1}\right)/{\text{ρ}}_{m}\right|<\text{ε}$$

It is assumed that a level of calculation error (ε) is better than 0.08%.

A coincidence is assumed to be within the 2.8%. The main drawback of all one-electron calculations consists in an underestimation of the band gap magnitudes [13]. For this reason, self-energy correction renormalization including scissor fitting factors [14] was included in the calculations. The local electronic structure of the reconstructed MgB_2_ was obtained using quantum chemical simulations within a framework of semi-empirical approach in the different standard quantum chemical package.

The effective total potential was built as a superposition of reconstructed background (due to relaxed MgB_2_ and external effective electric field) and perfect long-range order contribution:

(7) $$V\left(G\right)={\left(\frac{1}{\Omega_{cr}}\right)}_{am}\int_{cr}V^{cr}\left(r\right)\exp\left(iGr\right)d^{3}r+\left(\frac{1}{\Omega_{am-grain}}\right)\int_{am-grain}V^{am-grain}\left(r\right)\exp\left(iGr\right)d^{3}r$$

where **G** defines effective plane-wave vector within the first BZ of the perfect crystal; *g_am_* and *g_cr_* correspond to the weighting factors directly connected to the occurrence of partial amorphization and *V*^am^(*r*) and *V*^cr^(*r*) are pseudopotentials corresponding to the reconstructed and perfect crystallite states, respectively.

To accelerate the self-consistent procedure for determining the eigenvalues, we have modified the initial norm-conserving PP wave functions modified through their orthogonalization with respect to LCAO wave functions as described in the Reference [15]. Additionally, contributions of the anharmonic phonons were considered [16].

### 2.2. Structural Optimization

**Figure 1:**
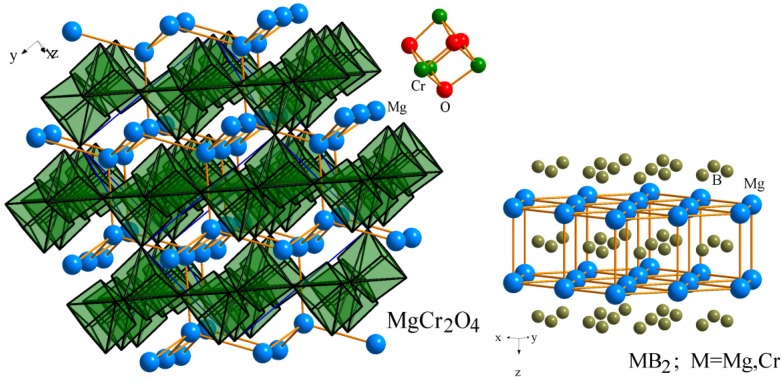
Laying atoms and some fragments in the structure or chromium magnesium diboride and MgCr_2_O_4_.

The problem of fusion between magnesium diboride layers and chromium atoms is still not completely clear. In the paper [5], possible mechanisms of such process were discussed based on the analysis of inter-atomic distances. To perform the mentioned structural optimization we have used the basic structural results given in the references [17,18,19]. Following the classical molecular dynamics simulations, a formation of intermediate MgCr_2_O_4_ crystalline structure is more likely [20]. The basic MgCr_2_O_4_ can identify defective deformed hexagonal and hexagonal 3D frame of atoms Mg (Figure 1). This structure was presented as partially disturbed by perfect structure of MgB_2_. We have assumed that an oxygen intermediate layer may occur during the mentioned fusion on the border. The thickness of the oxide layer will essentially depend on the oxygen content of the atmosphere during the deposition.

We have applied the above-described band structure procedure both for the basic MgB_2_ as well as to MgCr_2_O_4_ phase. As a consequence, the total energy deviation compared to the energy cut-off and the Perdew-Alder screening potential coefficients [21] was stabilized within the magnitudes equal to about 0.11 eV. It is interesting that at the beginning we have carried out the total energy minimization within a framework of the LDA pseudopotential approach [22]. However, it was established that this approach was non-appropriate for the total energy minimization of particular clusters, especially in the case of bulk symmetry breaking due to addition of another phase [23].

This case was similar to the substantially disordered materials like spin glasses or organic materials. In this case, it was important to do additional total energy minimization for such formed hybrid clusters using the space derivative of the potentials on the boundaries separating different phases. The total energy of the reconstructed cluster was assumed to be a simple statistic sum renormalized by appropriate Boltzmann weighting factor.

For simulation of the local long-range ordering existed in the basic MgB_2_ films, additional calculations of the total energy for the perfect and partially reconstructed phases were done. The simulations were limited by up to five to six coordination layers containing a mixture of MgB_2_ and MgCr_2_O_4_. The local cluster geometry of initially perfect crystal was modified by application of quantum chemical package HYPERCHEM-7. The starting wave functions were evaluated following atomic and total energy potential minimization.

In the case of a partially-disordered system, we have used a variation approach with Green function basis. This one allowed a reconstruction of the structure of the titled films and of the surrounding background [24].

The optimization procedure has been begun from sheets containing both phases of the mentioned crystallitnes. This sheet was considered like a nano-sheet because its sizes were varied within the 2–9 nm. At this stage a varying fitting parameter of NP sizes was introduced. The iteration procedure has been initiated from the long-range ordered MgB_2_ region and was successively continued for the disordered region.

The molecular dynamics optimization at the first stage was done until the total energy minimum magnitude for a fixed cluster was almost the same as for the earlier established for the case of long-range ordered crystal. The total energy for the cluster was presented as a partial sum of the structural clusters with appropriate weighting factors corresponding to contributions of the interfaces. At the second stage, the next layer is taken into account and the iteration procedure was repeated for the next iteration step. The above-described approach was performed iteratively until the relative displacement of atoms from their positions was less than 0.24 Å.

### 2.3. Principal Approaches for Simulation of Electronic Structure in the Reconstructed MgB_2_:Cr_2_O_3_ Nano-Interfaces

From the previous paragraph it is clear that crucial role in the observed dependences concerning the optical and electronic properties play reconstructed interface sheets with nano-sizes, which possess the thickness equal to about 2–9 nm. In all the nano-clusters and particularly in the large-sized ones (higher than 10 nm), the principal parameter determining manifestation of the nano-confined effect is a ratio between the thickness of the sheet reconstructed and of the total nanoparticle diameter. The problem of the reconstructed surfaces in such kinds of the materials consists of several ambiguities of using the principles of equilibrium thermodynamics, because the formed structures are sensitive to technological conditions.

So one can assume an occurrence of reconstructed near-the-surface structure possessing structural clusters with conformations corresponding to thermodynamically metastable (or even unstable) states. Usually, a structural optimization of the clusters with inclusion of different molecular dynamics method consists in variation of principal structural parameters (bond lengths, angles, torsion angles, *etc.*) to build a structural conformation defined by a minimum of total energy. For the case of the large-sized nano-crystallites (sizes about 20 nm) we assume an existence of the bulk-like perfect crystallites because principles of long-range ordered translation symmetry described by the Bloch rule are applicable. However, the long-range translation symmetry is broken near the interfaces. For this reason, Reference [10] proposed an effective approach combining equilibrium thermodynamics with non-equilibrium perturbation approaches formed by surrounding disordered background.

The method may be applicable for different condensed matter and considers a superposition of several structural fragments renormalized by appropriate weighting factors. The main approach consists of choosing of a few coordinated layers (usually with sizes which did not exceed 6 nm) having perfect long-range translation symmetry. The geometry optimization was carried out between the ordered layer and disordered ones with fixed atomic positions for the crystalline layers.

The reconstruction of the first layer is achieved through a variation of atomic positions. However, we have at least 4–6 layers for perfect long-range ordered crystal (which are fixed) and only addition (mixture) of one disordered (perturbed) layer, which is under modification. We obtained the first modified layered structure in a form of slightly modified perfect crystalline structure. At the next step, this modified layer was added to the perfect crystal structure and the iteration was repeated for the next layer. The total energy here was considered as a varied parameter. Main criterion consisted of achievement of a minimum of the total energy with stabilization up to 0.11 eV. To avoid potential jumps on the borders we carried out an additional derivative procedure. Different approaches of molecular dynamics were verified here, and Becke’s method was more appropriate. One of advantages of this method is an opportunity to include also dipole-dipole interactions between the particular clusters for the chosen cluster. Finally, we have obtained a structural cluster containing 50–70 atoms which were equivalent to clusters with 4–7 layers of the reconstructed interfaces.

## 3. Results and Discussions

### 3.1. Band Structure Calculations

Following the performed structural optimization it was established that both static as well as transition dipole moments of the reconstructed layer should play a prevailing role in the effects observed. We have established that the valence band for the MgB_2_ was mainly formed by 2 pB states. The higher dispersion confirms anisotropy of the chemical bonds in the BZ directions Γ–K. Generally, the obtained bands are close to the quasi-two dimensional type and they are flatter in the Γ–A BZ direction.

**Figure 2:**
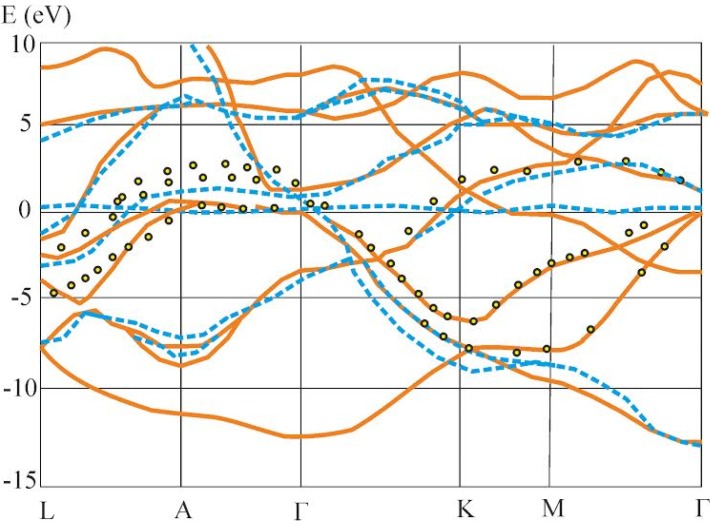
A typical fragment of the MgB_2_ calculated band structure with addition of the Cr_2_O_3_ (dotted line) and the superposition of the bicolor laser beams at 10.6 μm at power density about 0.8 GW/cm^2^. For the convenience of the readers only BZ directions with maximal deviation from the perfect MgB_2_ structure are presented.

Using appropriate weighting factors for the structural form-factors, the band energy dispersion of perfect crystalline states with reconstructed interfaces was calculated. It is shown in Figure 2. First of all, one can observe an occurrence of substantially flatter energy dispersion in the ***k***-space. The origin of such flattening is transformation from the delocalized collective states.

As a basic quasi-Brillouine zone we chose a perfect MgB_2_ crystalline structure. We present only the bands which are more sensitive to the changes during transformation from the perfect to the partially disordered states.

Additionally, we have superimposed the effective electric field which was equivalent to the photoexcitation by the two coherent beams used in the reference [17]. The changes of the effective electronic charge density due to photoexcitaion also were taken into account. Both electronic as well as phonon excitations following the reference [25] were taken into account. One can see that the superposition of the external laser beam could also lead to the some energy shift of the bands in the L–A–Γ direction. The discrete-like behavior for the band energy dispersion in the space favors also an occurrence of higher dipole moments determining principal optical susceptibility dispersion. Following Figure 2 one can see that principal deviations are observed for the BZ direction Γ–K–M. Such deviations show a possibility to take into account the contribution of the local disturbances which normally is not possible for the other types of the methods.

It is clear that the flattening in *k*-space of the band energy dispersion will be higher in the direction of the L–A–Γ, where exists stronger interaction between the basic MgB_2_ matrix and Cr_2_O_3_. So, choosing the structural component of the long-range crystallites corresponding to a maximal structural content, one can achieve an appearance of the varying band energy dispersion during moving from the border into the depth of the disordered structure. Moreover, it may be presented as a coexistence of several superstructures with a modulation wave vector substantially varying the space derivative of the potential possessing re-normalized ground and transition dipole moments. The latter one defines the enhanced output susceptibilities (both linear and non-linear ones) due to higher dipole moment’s magnitudes on the boundaries.

The determination of structural form-factor is carried out following a procedure of superposition of different structural phases with appropriate weighting factors.

### 3.2. Nonlinear Optical Dispersion

In the previous part we have shown substantial role of the optimized cluster in the modification of their band structure dispersion. However, NLO effects should be more important for optoelectronic and quantum electronics. It is caused by the fact that excited states (conduction sub-bands) should play a dominant role in this case. So, by modifying key structural parameters in a desirable direction, one can operate by the photoinduced superconductivity.

Local hyperpolarizability may achieve relatively large values; however, the relation between such microscopic hyperpolarizability and macroscopic electro-optic coefficient is difficult to evaluate from the first principles. Here, it should be mentioned that the hybrid organic-inorganic electrooptics have an advantage relative to inorganic crystals, liquid crystalline and organic chromospheres.

Effective SHG coefficients were evaluated using the acentric order parameter—<cos^3^θ> and an expression:

(8) $$r_{ijk}=2\beta_{ijk}N<\cos^{3}\left(\theta \right)>L_{ijk}/n^{2}$$

where *N* is a number of actual chromophore per volume unit; *n* is effective refractive index. During neglecting of inter-dipolar nano-cluster interaction the order parameter was described by equation:

(9) $$<\cos^{3}(\theta )>=\mu E_{eff}/k_{B}T$$

where μ is ground state dipole moment determining by reconstructed surfaces and surrounding modified polymer sheets. Very useful here was a value of μ*E_eff_*. From the presented equations it is clear that the second-order optical susceptibilities should increase linearly with the nanocluster’s numbers. However, at higher concentration of inter-molecular interactions *IM*, Equation (10) should be used.

(10) $$IM=\mu_{NC}\mu_{pol}/R^{3}$$

Here, *R* is a distance between the nanoclusters possessing state dipole moments μ*_NC_* and surrounding background with dipole moment μ*_pol_*.

The second order suscpetilbities for two principal components with repsect to the external light polarization *z* were determined using the follwong equations:

(11) $$\begin{array}{l} \chi_{Z⊥⊥}\left(-2\omega ;\omega ,\omega \right)=\frac{NL\left(2\omega \right)L^{2}\left(\omega \right)}{8\varepsilon_{0}}\left[\begin{array}{l} 2\beta_{zzz}\left(-2\omega ;\omega ,\omega \right)\langle \cos\theta -\cos^{3}\theta \rangle \\ +\sum_{i=x,y}\beta_{zii}\left(-2\omega ;\omega ,\omega \right)\langle \cos\theta -\cos^{3}\theta \rangle \\ -\sum_{i=x,y}2\beta_{iiz}\left(-2\omega ;\omega ,\omega \right)\langle \cos\theta -\cos^{3}\theta \rangle \end{array}\right]\\ \chi_{ZZZ}\left(-2\omega ;\omega ,\omega \right)=\frac{NL\left(2\omega \right)L^{2}\left(\omega \right)}{4\varepsilon_{0}}\left[\begin{array}{l} 2\beta_{zzz}\left(-2\omega ;\omega ,\omega \right)\langle \cos\theta -\cos^{3}\theta \rangle \\ +\sum_{i=x,y}\beta_{zii}\left(-2\omega ;\omega ,\omega \right)\langle \cos\theta -\cos^{3}\theta \rangle \\ +\sum_{i=x,y}2\beta_{iiz}\left(-2\omega ;\omega ,\omega \right)\langle \cos\theta -\cos^{3}\theta \rangle \end{array}\right] \end{array}$$

where *L*(ω) are *L*(*2*ω) are local field Lorentz factors, the brackets < > mean integration over the space orientation distribution of the molecules and the β*_ijk_* is the first order hyperpolarizability.

**Figure 3:**
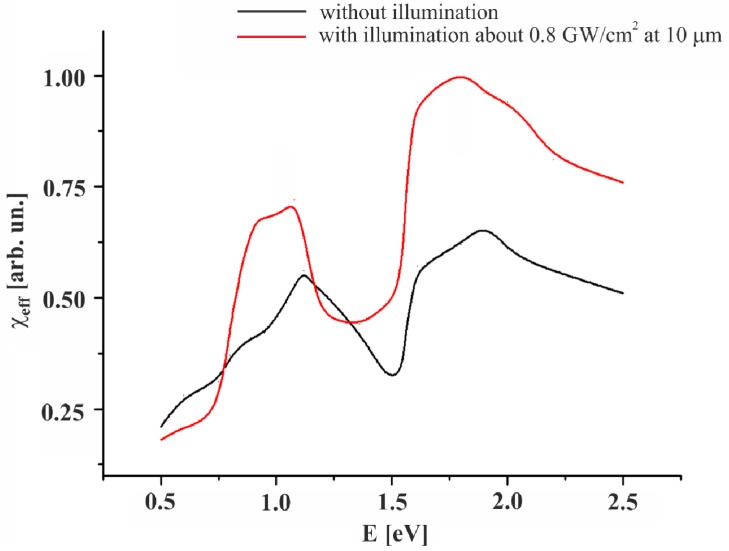
The calculated dispersion of the second order susceptibility with and without the laser stimulations.

The calculations of the second order susceptibility dispersion were done using a numerical method described in reference [26]. Following Figure 3 one can see that superposing of the external bicolor laser beams at 10.6 μm favors an enhanced second harmonic generation what was observed experimentally in the reference [9]. The two principal non-linear resonances at energies equal to about 1.1 eV and 1.7 eV give substantial rise under influence of external bicolor laser light. This fact is important because one can evaluate the partial contributions of the particular sub-bands states into the output susceptibilities. Another interesting factor was manifested in some red spectral shift of the observed nonlinear optical resonances (up to 0.2 eV). This asymmetry was enhanced with increasing photoinduced power density.

**Figure 4:**
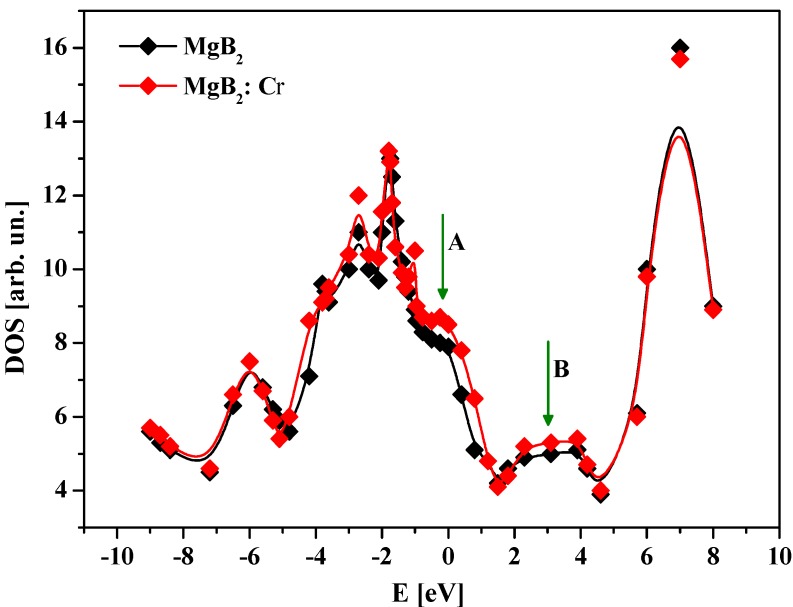
Density of electronic states for MgB_2_ and Cr doped MgB_2_.

**Figure 5:**
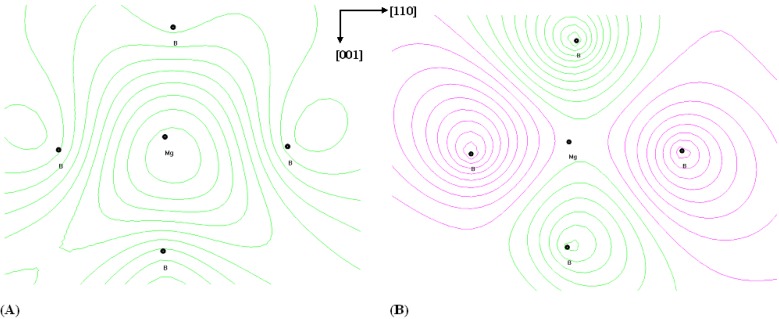
Charge density distribution for principal clusters of Mg-B_6_ for pure MgB_2_ films (**A**) and differential charge density for the Cr doped with respect to pure MgB_2_ films (**B**). Increment is equal to 0.2 e/Ω.

To show the principal strength of our method, we have performed studies of density of states for the MgB_2_ films and the Cr doped films (see Figure 4). Following this figure one can clearly see that principal changes occurred near the Fermi energy level (A) and excited states (at about 3–4 eV) (B). This was additionally confirmed by the space charge density distribution of the charge density for the principal (001–110) sequence of Mg-B_6_ structural fragments (see Figure 5). Even for the pure films one can see some charge density anisotropy in the (001–110) crystallographic plane. Addition of the Cr leads to higher space charge density anisotropy. This one is clear from the differential charge density for the Cr-doped and pure MgB_2_ films (see Figure 5). It was clearly different with respect to the TB-LMTO approach [27].

Taking into account the low-dimensional contributions, the same complications are typical for extended super-clustes [28]. The occurred charge density acentricity during the Cr doping confirmed the experimental results obtained by us concerning the nonlinear optical effects [5] as well as the observed laser induced infrared spectral shift [25].

The comparison of the charge density distribution for the principal sequence of the MgB_2_ oriented films with respect to the other calculations have clearly shown an occurrence of a slight charge density asymmetry which was absent during other types of calculation. Moreover, additional presence of the Cr ions leads to the additional charge density acentricity. It is clearly shown in Figure 5B, where the difference of the charge densities are presented for the pure and Cr doped MgB_2_. The obtained charge density acentricity may be a main source of the observed second order susceptibility.

All the former approaches for MgB_2_ [29,30,31] including for the doped [32] yield only slight influence on the symmetry. In our approach, we showed an occurrence of some charge density acentricity (see Figure 5). Therefore, it should play a principal role in the optically stimulated second order optical dispersion and is in agreement with the corresponding experimental data [17,25].

## 4. Conclusions

For the first time, first principal band structure calculations of the photoinduced changes of nonlinear optical properties in the high temperature superconductor films MgB_2_:Cr_2_O_3_ were performed. We found an occurrence of substantially flatter energy dispersion in the ***k***-space. The origin of the flattening is originated from transformation of the delocalized collective states to the more localized band states with larger effective masses. It is clear that the flattening in *k*-space of the band energy dispersion will be higher in the direction of the L–A–Γ where there exists more interaction between the basic MgB_2_ structural fragments and Cr_2_O_3_. So, choosing the structural component of the long-range crystallites corresponding to a maximal structural content, one can see an appearance of the varying band energy dispersion while moving from the border into the depth of the disordered structure. Moreover, it may be presented as a coexistence of several superstructures with a modulation wave vector substantially reorganizing the space derivative of the potential and re-normalized ground and transition dipole moments. The latter defines the enhanced output susceptibilities (both linear and non-linear ones) due to higher dipole moments on the boundaries.
